# Determinants of non-attendance at face-to-face and telemedicine ophthalmic consultations

**DOI:** 10.1136/bjo-2022-322389

**Published:** 2023-05-22

**Authors:** Siegfried K Wagner, Laxmi Raja, Mario Cortina-Borja, Josef Huemer, Robbert Struyven, Pearse A Keane, Konstantinos Balaskas, Dawn A Sim, Peter B M Thomas, Jugnoo S Rahi, Ameenat Lola Solebo, Swan Kang

**Affiliations:** 1 Institute of Ophthalmology, University College London, London, UK; 2 NIHR Moorfields Biomedical Research Centre, Moorfields Eye Hospital NHS Foundation Trust, London, UK; 3 Digital Clinical Laboratory, Moorfields Eye Hospital NHS Foundation Trust, London, UK; 4 Great Ormond Street Institute of Child Health, University College London, London, UK; 5 Department of Medical Retina, Moorfields Eye Hospital NHS Foundation Trust, London, UK; 6 Centre for Medical Image Computing, University College London, London, UK; 7 NIHR Biomedical Research Centre for Ophthalmology, Moorfields Eye Hospital NHS Foundation Trust and UCL, London, UK; 8 Department of Ophthamology, Great Ormond Street Hospital NHS Foundation Trust, London, UK; 9 Ulverscroft Vision Research Group, University College London, London, UK; 10 Adnexal department, Moorfields Eye Hospital NHS Foundation Trust, London, UK

**Keywords:** Telemedicine, Public health, Covid-19

## Abstract

**Background/aims:**

Evaluation of telemedicine care models has highlighted its potential for exacerbating healthcare inequalities. This study seeks to identify and characterise factors associated with non-attendance across face-to-face and telemedicine outpatient appointments.

**Methods:**

A retrospective cohort study at a tertiary-level ophthalmic institution in the UK, between 1 January 2019 and 31 October 2021. Logistic regression modelled non-attendance against sociodemographic, clinical and operational exposure variables for all new patient registrations across five delivery modes: asynchronous, synchronous telephone, synchronous audiovisual and face to face prior to the pandemic and face to face during the pandemic.

**Results:**

A total of 85 924 patients (median age 55 years, 54.4% female) were newly registered. Non-attendance differed significantly by delivery mode: (9.0% face to face prepandemic, 10.5% face to face during the pandemic, 11.7% asynchronous and 7.8%, synchronous during pandemic). Male sex, greater levels of deprivation, a previously cancelled appointment and not self-reporting ethnicity were strongly associated with non-attendance across all delivery modes. Individuals identifying as black ethnicity had worse attendance in synchronous audiovisual clinics (adjusted OR 4.24, 95% CI 1.59 to 11.28) but not asynchronous. Those not self-reporting their ethnicity were from more deprived backgrounds, had worse broadband access and had significantly higher non-attendance across all modes (all p<0.001).

**Conclusion:**

Persistent non-attendance among underserved populations attending telemedicine appointments highlights the challenge digital transformation faces for reducing healthcare inequalities. Implementation of new programmes should be accompanied by investigation into the differential health outcomes of vulnerable populations.

**WHAT IS ALREADY KNOWN ON THIS TOPIC**

- There is a growing evidence that digital transformation of healthcare services may be exacerbating healthcare inequalities. Patients who miss multiple hospital appointments, or ‘non-attenders’, are an under-researched group who may be suffering from substantial unmet health needs. Reports revealed a consistent relationship between reduced uptake of telemedicine appointments and greater levels of socioeconomic deprivation, low-income and ethnic minority groups however few examined non-attendance rates.

**WHAT THIS STUDY ADDS**

- In this cohort study across 86 049 patients, non-attendance in synchronous audiovisual appointments was highest among men, those from greater levels of deprivation, those experiencing a previously cancelled appointment and those not self-reporting their ethnicity.

**HOW THIS STUDY MIGHT AFFECT RESEARCH, PRACTICE OR POLICY**

- Persisting disparities in healthcare engagement among certain sociodemographic groups risks exacerbating pre-existing inequalities. Development of telemedicine services should go hand in hand with investigations into differential health outcomes among underserved populations.

## Introduction

In response to the COVID-19 pandemic, telemedicine became requisite to maintaining eye care delivery, with deployment across different nations.^1–6^ Implemented at speed, and without an evidence base to inform mitigating strategies to prevent digital exclusion, there was a risk that greater reliance on digital technology could compound existing health disparities based on accessibility to and engagement with digital tools.^7–9^ Emerging evidence suggests this may be occurring—one US-based study found patients of older age and from ethnic minority groups were less likely to complete a teleophthalmology appointment.^10^ Similar patterns have been seen in electronic health record patient portal systems.^11 12^ Whether such disparities reflect an exacerbation of pre-existing inequalities or simply echo those found in traditional office-based consultations remains unclear. Moreover, most findings thus far have been derived from systems where the financial costs of access may influence healthcare engagement. Little attention has also been given to asynchronous ‘store-and-forward’ teleophthalmology approaches, an increasingly popular model of healthcare delivery.^13^

Moorfields Eye Hospital (MEH) is the largest tertiary ophthalmic centre based in the UK, providing eye services to an ethnically and socioeconomically diverse catchment population of approximately six million people in London, UK through both telemedicine (asynchronous and synchronous) and face-to-face encounters. In this study, our primary objective was to identify sociodemographic, clinical and operational factors associated with non-attendance at telemedicine clinics in specialist ophthalmic care within the National Health Service (NHS) which provides cost-free care at the point of use. We hypothesised that those from ethnic minority groups, or living with greater socioeconomic deprivation, or with limited internet access would have higher levels of non-attendance at synchronous telemedicine clinics. We additionally compared non-attendance between asynchronous, synchronous clinic delivery modes (collectively termed telemedicine) and face-to-face clinics.

## Methods

This was a retrospective cohort design of all NHS patients, aged 18 and over, who were newly registered and referred to MEH, between 1 January 2019 and 31 October 2021 inclusive. Only attendance or non-attendance at the first appointment at MEH was analysed.

Patients previously registered at MEH were excluded. We included patients referred to the adnexal, cataract, general ophthalmology, glaucoma and medical retina services as these accounted for 98.0% of all virtual clinics at that time. Sociodemographic, clinical variables and type of appointment were extracted from the MEH data warehouse, a locally held central repository of aggregated data from all electronic health record systems. Ethnicity was self-reported by the patient as (1) Asian or Asian British, (2) black or black British, (3) mixed, (4) other ethnic group, (5) white or (6) unknown. Due to data sparsity, those identifying as mixed were aggregated with other ethnic group. Socioeconomic status (SES) was measured using the Index of Multiple Deprivation (IMD) 2019, a standard UK measure of relative deprivation and SES across seven domains of income, employment, education, health and barriers to housing and services, crime and living environment.^14^ Access to and speed of home broadband internet was derived from the Digital Exclusion Risk Index (DERI), a composite continuous score between 1 and 10 developed by Greater Manchester Combined Authority and the Good Things Foundation.^15^ Due to small numbers resulting in potential loss of anonymity and limited statistical powers, patients certified as being sight impaired or severely sight impaired (equivalent to severe visual impairment or blindness using WHO criteria and conferring Government assistance) were aggregated into a single group.

Our primary outcome was attendance at the first appointment, defined as a binary variable. During the period studied, 139 908 appointments were cancelled by either the hospital or patient. As our study period included the start of the pandemic, the reason for cancellation was not consistently available and we were interested in identifying determinants of non-attendance, we used a previously cancelled appointment as an exposure variable and further classified whether it was instigated by the patient or by the hospital. Thus, the following exposure variables were defined a priori based on literature review and other hypothesised reasons for non-attendance^16–20^:

- Sociodemographic—age (continuous), biological sex (binary), ethnic group (categorical), SES (rank), interpreter requirement (binary), broadband access (continuous).
- Clinical—diabetes mellitus (binary), ophthalmic subspecialty (categorical), certificate of visual impairment registration (binary).
- Operational—appointment time (categorical of early morning (8:00–11:00 hours), late morning (11:00–13:00 hours), early afternoon (13:00-15:00 hours) and late afternoon (15:00–17:00 hours)), previous cancellation by the hospital, previous cancellation by the patient.

Appointments were categorised by mode of delivery into one of the following three main forms of contact between the patient and clinician planning treatment:

- A store-and-forward approach where patients attend in person and undergo assessment with subsequent remote review by a clinician (hereafter termed ‘asynchronous’). Outcome of the appointment is typically communicated to the patient through postal letter. Rarely, for urgent sight-threatening or life-threatening pathology, the patient may be contacted by the healthcare professional by telephone.
- A live technique mode where a clinician interacts in real-time with patients either through telephone or a audiovisual means (hereafter termed ‘synchronous’).
- Traditional face-to-face attendance with real-time interaction with a clinician (hereafter termed ‘F2F’).

Our primary objective was to evaluate the determinants of non-attendance at telemedicine appointments, comparing asynchronous and synchronous. Separately, we evaluated the determinants of non-attendance at F2F appointments for the same time period but also, for ‘benchmarking’ non-attendance at F2F appointments before the pandemic, that is, from 1 January 2019 to the first UK lockdown on 23 March 2021.

### Statistical analysis

Continuous variables are summarised as median±IQR and categorical variables through percentages. Categorical variables were compared using the U-statistic permutation test of independence^21^ and continuous variables through the Wilcoxon-Mann-Whitney U test and Kruskal-Wallis Test. Individual pairwise comparisons were through the Dunn method with correction for multiple testing using the Bonferonni-Holm procedure.

Handling of missing data is reported according to recommendations issued on behalf of the STRengthening Analytical Thinking for Observational Studies initiative.^22^ There was a substantial number of missing data for self-reported ethnicity (n=53 864, 62.6%). We assumed that ethnicity data were not missing completely at random (MCAR) based on previous evidence of the sociodemographic determinants of missingness on self-reporting in healthcare.^23–25^ Moreover, there was strong statistical evidence to reject the null hypothesis that the data was MCAR using Little’s test (p<0.001). In our primary analysis, we hypothesised lack of self-reporting of ethnicity to be an important surrogate of altered engagement with health services and therefore separately modelled unreported ethnicity as a specific category, ‘unknown’, cognisant that this could shift any measures of effect for ethnic minority groups towards neutrality. Nonetheless, we analysed baseline characteristics among those who did not self-report ethnicity against those who did and, as a sensitivity analysis, we performed conditional multiple imputation 10 times with 5 iterations using multinomial logistic regression using all other exposure variables, in their raw form. Apart from self-reported ethnicity, no other variable had a large proportion of missingness (all <1%).

Adjusted ORs (aOR) with 95% CIs were estimated from multivariable binomial logistic regression using attendance status as the dependent variable and stratified by delivery mode. Five final models, fitted to all a priori exposure variables, were constructed depending on delivery mode (asynchronous, synchronous telephone, synchronous audiovisual, F2F and F2F in the year before the pandemic).

## Results

**Figure 1:**
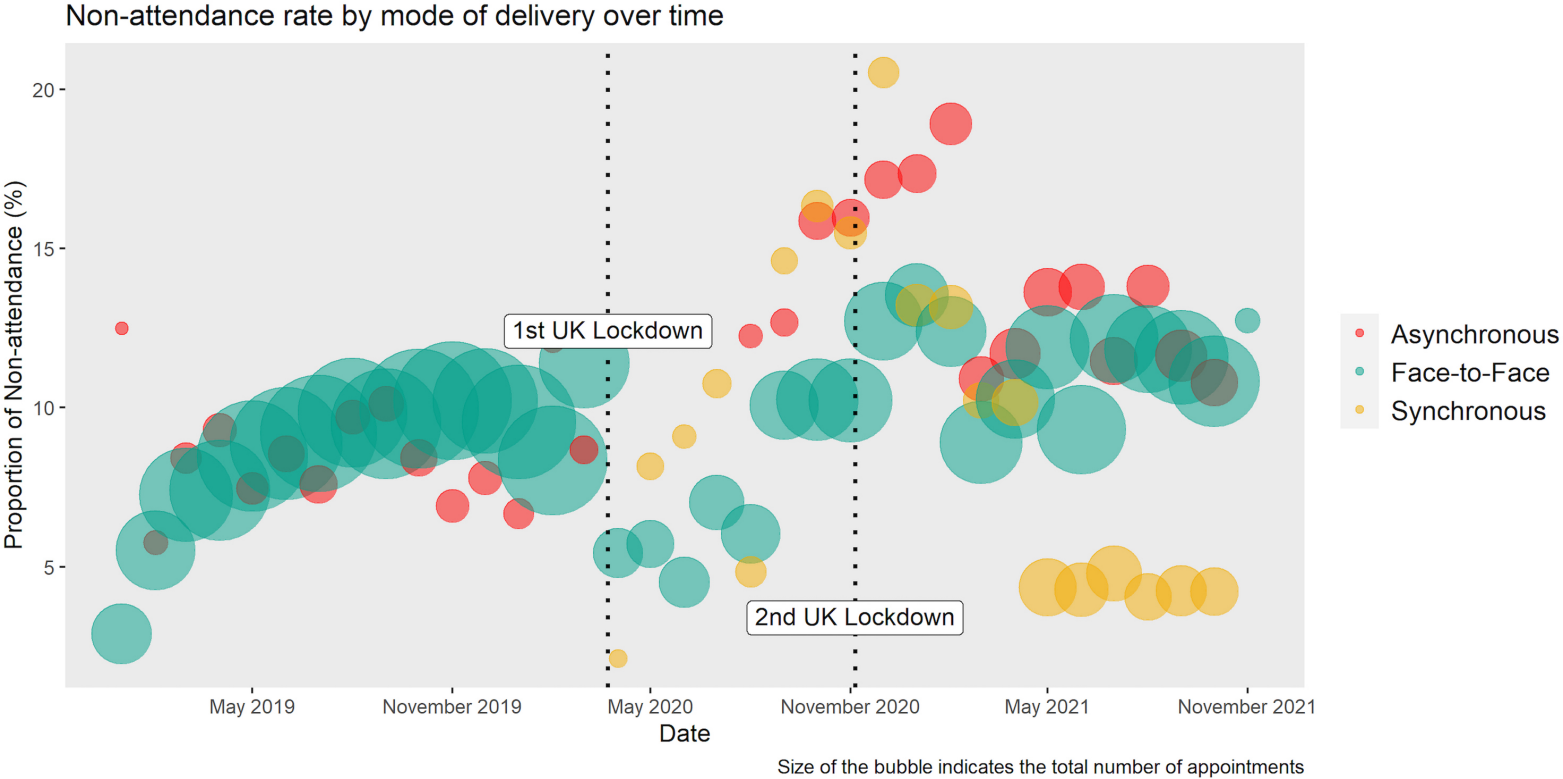
Bubble plot showing the proportion of non-attendances for newly registered and referred patients during the time period. Size of the bubble indicates the number of patients.

Between 1 January 2019 and 31 October 2021, 85 924 patients were newly registered and referred to services across all MEH sites (70 328 F2F, 8878 asynchronous and 6718 synchronous (online supplemental figures 1 and 2). Change in non-attendance rates over the study period are shown in figure 1. Median age of the cohort was 55±15 years and 54.4% (n=46 795) were female. Patients receiving their first appointment through synchronous audiovisual were the youngest (median 39±12.5 years) whereas those undergoing asynchronous review were older (median 57±10 years, p<0.001, table 1). Further baseline characteristics by delivery mode can be found in table 1. Individual pairwise comparisons among the delivery modes for age, sex, ethnicity, SES and non-attendance are in online supplemental table 1. Individuals who did not self-report ethnicity were more likely to be female, older, have diabetes mellitus and experience greater levels of socioeconomic deprivation and worse broadband access (online supplemental table 2).

10.1136/bjo-2022-322389.supp1 Supplementary data

**Table 1:** Baseline characteristics by delivery mode

Category	F2F prepandemic n=42 972	F2F pandemic n=27 356	Asynchronous n=8878	Synchronous telephone n=1480	Synchronous audiovisual n=5238	P value*
Sex n (%)						
Female	23 430 (54.5)	14 773 (54.0)	4620 (52.0)	845 (57.1)	3068 (58.6)	<0.001
Age median (IQR)						
Years	54 (30)	56 (30)	57 (20)	68 (19)	39 (25)	<0.001
Ethnicity n (%)						
Asian	2389 (5.6)	1208 (4.4)	270 (3.0)	58 (3.9)	175 (3.3)	<0.001
Black	1365 (3.2)	546 (2.0)	290 (3.3)	23 (1.6)	71 (1.4)	
Other	8054 (18.7)	5461 (20.0)	743 (8.4)	125 (8.5)	1596 (30.5)	
White	5835 (13.6)	2613 (9.6)	802 (9.0)	66 (4.5)	447 (8.5)	
Unknown	25 329 (58.9)	17 528 (64.1)	6773 (76.3)	1208 (81.6)	2949 (56.3)	
SES† median (IQR)						
Decile (1=most deprived)	5 (4)	5 (4)	5 (4)	5 (4)	4 (3)	<0.001
Broadband access‡ median (IQR)						
Index (1=most at risk of digital exclusion)	3.40 (0.44)	3.42 (0.47)	3.41 (0.43)	3.41 (0.45)	3.42 (0.47)	0.008
Interpreter n (%)						
Yes	406 (0.9)	214 (0.8)	69 (0.8)	14 (0.9)	29 (0.6)	0.007
Diabetes n (%)§						
Yes	1824 (4.2)	1454 (5.3)	887 (10.0)	17 (1.2)	10 (0.2)	<0.001
Time of appointmentn (%)						
Early morning	20 115 (46.8)	12 619 (46.1)	3618 (40.8)	396 (36.8)	1815 (34.7)	<0.001
Late morning	2872 (6.7)	2426 (8.9)	1354 (15.2)	129 (8.7)	679 (13.0)	
Early afternoon	16 298 (37.9)	9990 (36.5)	2502 (28.2)	644 (43.5)	1589 (30.3)	
Late afternoon	3687 (8.6)	2321 (8.5)	1404 (15.8)	311 (21.0)	1155 (22.1)	
Sight-impaired n (%)						
Yes	266 (0.6)	189 (0.7)	30 (0.3)	¶	¶	<0.001
Previous cancellation n (%)						
No	36 477 (84.9)	22 744 (83.1)	7228 (81.4)	1138 (76.9)	4675 (89.3)	<0.001
By hospital	4650 (10.8)	3482 (12.7)	925 (10.4)	275 (18.6)	448 (8.6)	
By patient	1845 (4.3)	1130 (4.1)	725 (8.2)	67 (4.5)	115 (2.2)	
Specialty n (%)						
Adnexal	3278 (7.6)	2441 (8.9)	**	129 (8.7)	1174 (22.4)	<0.001
Cataract	7782 (18.1)	6017 (22.0)	**	1171 (79.1)	149 (2.8)	
General	20 093 (46.8)	11 025 (40.3)	**	180 (12.2)	3915 (74.7)	
Glaucoma	3883 (9.0)	1955 (7.2)	6138 (69.1)	**	**	
Medical retina	7936 (18.5)	5918 (21.6)	2740 (30.9)	**	**	
Attendance status n (%)						
Non-attendance	3860 (9.0)	2868 (10.5)	1042 (11.7)	145 (9.8)	373 (7.1)	<0.001

*P values derived from U-Statistic permutation test for categorical variables and Kruskal-Wallis test for continuous variables looking at differences between all groups. For individual pairwise comparisons, see online supplemental table 1.

†Missing values from 216 patients. A lower number equates to greater deprivation.

‡Missing values from 216 patients. A lower number equates to a higher risk of digital exclusion.

§Footnote reminder here that medical retina is one of the two specialties that offered asynchronous care.

¶Figures suppressed due to small number of patients.

**No or minimal appointments for these subspecialties in this mode of delivery.

F2F, face to face; SES, socioeconomic status.

### Factors associated with non-attendance

Distribution of attendance status by exposure variables for all appointments can be seen in table 2. Overall non-attendance was 9.7% across all first appointments (n=8306). Non-attendance was highest in asynchronous clinics (11.7%) and lowest in synchronous audiovisual clinics (7.1%, table 1). Non-attendance was higher among younger patients, men, those experiencing a previously cancelled appointment and those from more socioeconomically deprived groups (all p<0.001, table 2). We did not find an association between interpreter requirement and non-attendance.

**Table 2:** Distribution of secondary exposure variables by attendance status

Characteristic	Attended (n=77 743)	Did not attend (n=8306)	P value
Age	53.5 (19.0)	51.5 (19.1)	<0.001
Sex			
Female	42 579 (91.0%)	4216 (9.0%)	<0.001
Male	35 164 (89.6%)	4090 (10.4%)	
Ethnicity			
Asian (South)	3827 (93.3%)	275 (6.7%)	<0.001
Black	2093 (91.1%)	204 (8.9%)	
Other	14 621 (91.5%)	1366 (8.5%)	
Unknown	47 932 (88.9%)	5962 (11.1%)	
White	9270 (94.9%)	499 (5.1%)	
Socioeconomic deprivation (decile)*	5 (4)	4 (3)	<0.001
Broadband access (index)	3.40	3.42	0.011
Subspecialty			
Adnexal	6322 (89.9%)	707 (10.1%)	<0.001
Cataract	13 747 (90.6%)	1431 (9.4%)	
General	32 661 (92.8%)	2553 (7.2%)	
Glaucoma	10 755 (89.8%)	1224 (10.2%)	
Medical retina	14 258 (85.6%)	2391 (14.4%)	
Interpreter required			
Yes	669 (91.3%)	64 (8.7%)	0.385
No	77 074 (90.3%)	8242 (9.7%)	
Diabetes mellitus			
Yes	3747 (89.3%)	449 (10.7%)	0.021
No	73 996 (90.4%)	7857 (9.6%)	
Time of appointment			
Early morning	34 519 (89.4%)	4109 (10.6%)	<0.001
Late morning	6879 (92.0%)	599 (8.0%)	
Early afternoon	28 031 (90.3%)	3025 (9.7%)	
Late afternoon	8314 (93.6%)	573 (6.4%)	
Registered sight-impaired			
Yes	469 (94.9%)	25 (5.1%)	<0.001
No	77 274 (90.3%)	8281 (9.7%)	
Previous cancellation			
No	66 012 (91.2%)	6365 (8.8%)	<0.001
By hospital	8435 (86.2%)	1354 (13.8%)	
By patient	3296 (84.9%)	587 (15.1%)	

*Missing values from 216 individuals (0.3% of the dataset).

**Figure 2:**
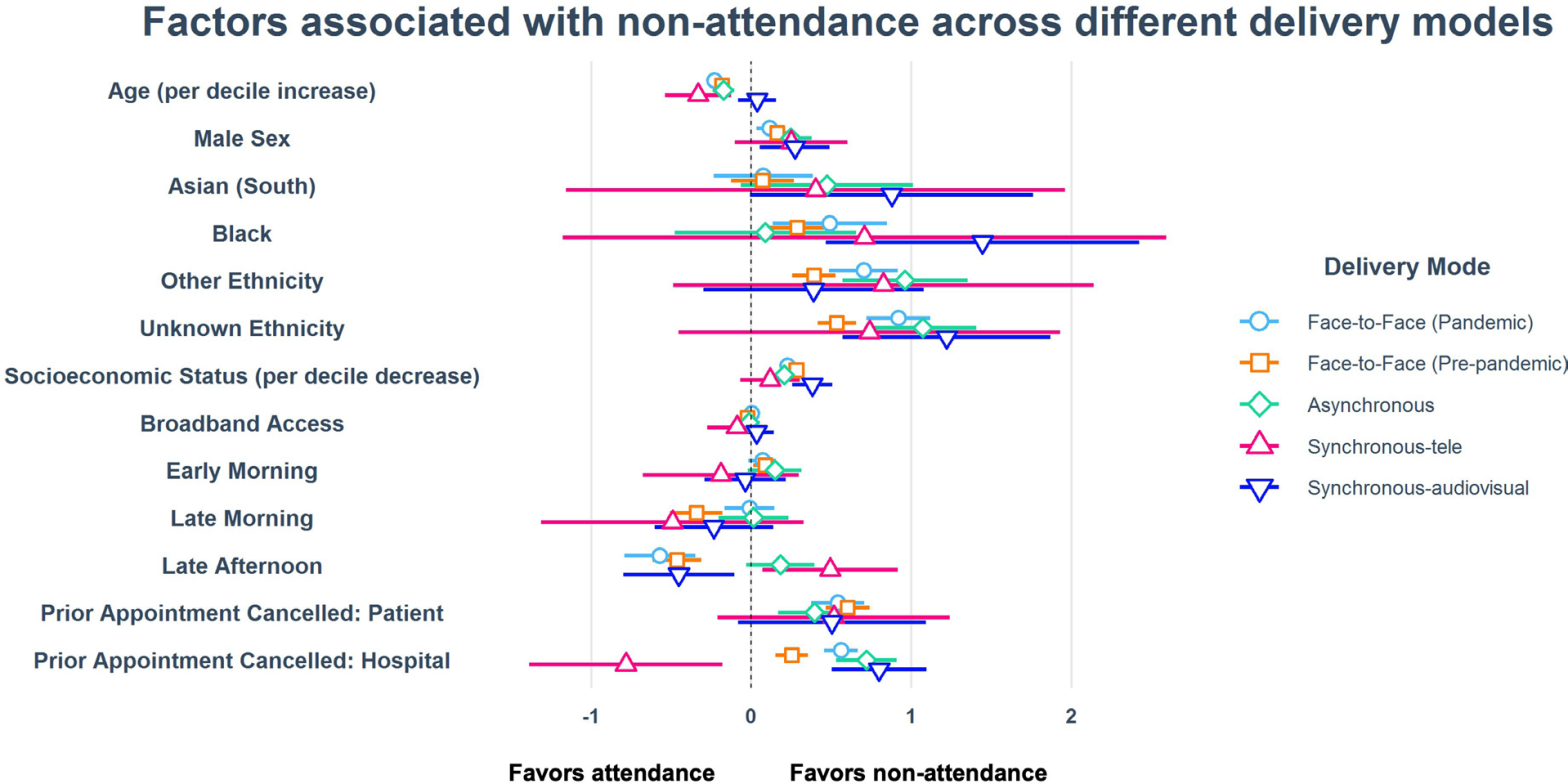
Forest plot showing regression coefficient estimates with 95% CIs by delivery mode derived from logistic regression. Note that sight-impairment registration, diabetes mellitus and subspecialty are not shown due to unstable estimates from small numbers.

aORs modelling non-attendance are shown in table 3 (further details in online supplemental table 3) and figure 2. Across all delivery modes, men, those with greater levels of deprivation (except synchronous telephone) and those with a previously cancelled appointment by the hospital had higher levels of non-attendance. Increasing age was associated with greater levels of attendance across F2F and asynchronous clinics but not with synchronous.

**Table 3:** Adjusted ORs for non-attendance derived from multivariable logistic regression stratified by delivery mode

	Prepandemic F2F	Pandemic F2F	Asynchronous	Synchronous (telephone)	Synchronous (audiovisual)
	OR (95% CI)	OR (95% CI)	OR (95% CI)	OR (95% CI)	OR (95% CI)
Age					
(Decade)	**0.91 (0.89 to 0.93**)	**0.89 (0.87 to 0.91**)	**0.89 (0.85 to 0.93**)	**0.81 (0.72 to 0.93**)	1.02 (0.95 to 1.09)
Sex					
Female	Reference	Reference	Reference	Reference	Reference
Male	**1.18 (1.10 to 1.26**)	**1.12 (1.03 to 1.21**)	**1.28 (1.12 to 1.46**)	1.28 (0.90 to 1.82)	**1.31 (1.06 to 1.63**)
Ethnicity					
White	Reference	Reference	Reference	Reference	Reference
Asian	1.07 (0.88 to 1.30)	1.08 (0.79 to 1.47)	1.60 (0.94 to 2.75)	1.49 (0.31 to 7.11)	2.40 (0.99 to 5.82)
Black	**1.33 (1.07 to 1.65**)	**1.63 (1.14 to 2.33**)	1.09 (0.62 to 1.92)	2.03 (0.31 to 13.37)	**4.24 (1.59 to 11.28**)
Other	**1.48 (1.29 to 1.70**)	**2.02 (1.63 to 2.50**)	**2.61 (1.77 to 3.86**)	2.28 (0.61 to 8.49)	1.48 (0.74 to 2.94)
Unknown	**1.71 (1.51 to 1.92**)	**2.51 (2.05 to 3.05**)	**2.92 (2.09 to 4.08**)	2.09 (0.64 to 6.89)	**3.39 (1.77 to 6.48**)
SES (greater deprivation)					
Per decile decrease	**1.12 (1.10 to 1.13**)	**1.09 (1.07 to 1.11**)	**1.09 (1.05 to 1.12**)	1.05 (0.97 to 1.13)	**1.17 (1.11 to 1.23**)
Broadband					
Index	0.94 (0.87 to 1.02)	1.00 (0.91 to 1.10)	0.97 (0.93 to 1.14)	0.81 (0.52 to 1.26)	1.07 (0.85 to 1.36)
Interpreter					
No	Reference	Reference	Reference	Reference	Reference
Yes	**0.53 (0.35 to 0.81**)	1.07 (0.70 to 1.63)	1.31 (0.66 to 2.60)	3.31 (1.00 to 10.96)	0.46 (0.01 to 3.39)
Diabetes mellitus					
No	Reference	Reference	Reference	Reference	Reference
Yes	0.87 (0.74 to 1.03)	**0.56 (0.47 to 0.68**)	**0.46 (0.36 to 0.59**)	1.41 (0.30 to 6.53)	*
Subspecialty					
Adnexal	Reference	Reference	*	Reference	Reference
Cataract	**1.51 (1.28 to 1.78**)	1.06 (0.91 to 1.23)	*	1.31 (0.60 to 2.83)	1.12 (0.67 to 1.87)
General	**1.37 (1.19 to 1.59**)	0.50 (0.43 to 0.58)	*	1.42 (0.64 to 3.13)	**0.70 (0.55 to 0.90**)
Glaucoma	**1.68 (1.41 to 1.99**)	1.00 (0.83 to 1.20)	Reference	*	*
MR	**2.08 (1.78 to 2.43**)	**1.48 (1.28 to 1.70**)	**2.55 (2.20 to 2.96**)	*	*
Certified sight-impaired					
No	Reference	Reference	Reference	Reference	Reference
Yes	**0.39 (0.22 to 0.70**)	**0.50 (0.28 to 0.89**)	0.66 (0.15 to 2.84)	*	*
Appointment time					
Early afternoon	Reference	Reference	Reference	Reference	Reference
Early morning	**1.09 (1.01 to 1.17**)	1.07 (0.98 to 1.17)	1.16 (0.98 to 1.37)	0.83 (0.51 to 1.35)	0.96 (0.75 to 1.24)
Late morning	**0.71 (0.60 to 0.83**)	0.99 (0.85 to 1.15)	1.01 (0.81 to 1.26)	0.61 (0.27 to 1.39)	0.79 (0.55 to 1.15)
Late afternoon	**0.63 (0.54 to 0.73**)	**0.56 (0.45 to 0.70**)	1.20 (0.97 to 1.48)	**1.64 (1.07 to 2.50**)	**0.64 (0.45 to 0.90**)
Previous cancellation					
No	Reference	Reference	Reference	Reference	Reference
By the hospital	**1.29 (1.16 to 1.43**)	**1.75 (1.58 to 1.94**)	**2.05 (1.70 to 2.48**)	**0.46 (0.25 to 0.84**)	**2.22 (1.65 to 2.98**)
By the patient	**1.83 (1.59 to 2.09**)	**1.72 (1.45 to 2.03**)	**1.48 (1.18 to 1.86**)	1.67 (0.81 to 3.46)	1.65 (0.92 to 2.97)

Effect estimates in bold were statistically significant (see online supplemental table 3 for more information)

*Either no cases or very few leading to unstable estimates.

F2F, face to face; MR, medical retina; SES, socioeconomic status.

In regard to teleophthalmology clinics, patients identifying as Black ethnicity were more likely to not attend a synchronous audiovisual appointment (4.24, 95% CI 1.59 to 11.28, p=0.0039). However, there was no association between Asian or Black ethnicity with attendance status in asynchronous clinics. Patients had 105% and 48% greater odds of not attending their asynchronous clinic appointment if it had been previously cancelled by the hospital or patient, respectively; for synchronous audiovisual clinics, a previous cancellation by the hospital was associated with 122% greater odds of them not attending their appointment (p<0.001).

Appointment time was an important factor for attendance at F2F appointments: prior to the pandemic, early morning appointments were associated with a greater level of non-attendance (aOR 1.09, 95% CI 1.01 to 1.17) compared with early afternoon. Conversely, late afternoon appointments were attended more frequently (aOR 0.63, 95% CI 0.54 to 0.73 prior to the pandemic and 0.56, 95% CI 0.45 to 0.70 since the pandemic). Interestingly, patients with diabetes mellitus had lower levels of non-attendance when looking at F2F means during pandemic (0.56, 95% CI 0.47 to 0.68) and in asynchronous teleophthalmology (0.46, 95% CI 0.36 to 0.59). Those who were sight-impaired also independently had lower levels of non-attendance both prior to (0.39, 95% CI 0.22 to 0.70) and during the pandemic (0.50, 95% CI 0.28 to 0.89). Patients requiring an interpreter were more likely to attend prior to the pandemic (non-attendance 0.53, 95% CI 0.35 to 0.81, p=0.0036) but this changed during the pandemic (1.07, 95% CI 0.70 to 1.63).

On sensitivity analysis, multiple imputation showed similar measures of effect across all variables; however, measures of effect for non-attendance among Black and South Asian ethnic groups were more extreme for synchronous audiovisual appointments and South Asian patients had greater non-attendance in asynchronous (1.44, 95% CI 1.01 to 2.04, p=0.0416, (online supplemental table 4).

## Discussion

From an analysis of 85 924 patients newly registered in a tertiary ophthalmic healthcare service in the UK between 1 January 2019 and 31 October 2021, we found non-attendance across all delivery modes to be associated with male sex, greater socioeconomic deprivation, lack of ethnicity self-reporting and previously cancelled appointments (instigated by the patient or hospital). Self-identified Black ethnicity was the factor most strongly associated with non-attendance at a synchronous audiovisual appointment. Our report demonstrates that even within healthcare systems free at the point of service, socioeconomic deprivation is a major challenge to engagement with digital transformation of services.

The results of this study must be considered in the context of its limitations. First, as in any observational epidemiological study, we cannot rule out residual confounding (eg, employment and accommodation status)—however, the IMD score of socioeconomic deprivation does encompass some relevant metrics. In regard to self-reported ethnicity, there was a significant amount of missing data. Individuals choosing not to self-report their ethnicity demonstrate reduced engagement with healthcare services and we sought to describe this effect by assigning a category of unreported ethnicity in our primary analysis. Given that individuals from ethnic minority groups are less likely to self-report their ethnicity and the high non-attendance rates among those who failed to self-report, differential misclassification bias is likely to have underestimated the aOR for non-attendance for these groups. Indeed, this hypothesis was supported by our supplementary analyses using multiple imputation, a technique which reduces bias even with large proportions of missingness.^26 27^ There were a large number of cancelled appointments during the study period (~15%), resembling that seen in the UK NHS during a similar time period.^28^ We were able to distinguish between those initiated by the patient versus those by the hospital, however, the reasons for cancellation were not available rendering any association with our outcome or other exposure variables unclear. While formal standard operating procedures were not in place at this time regarding suitable candidates for teleophthalmology, administrative and healthcare professionals are likely to have risk-stratified patients being offered synchronous teleophthalmology appointments resulting in a selection bias. Similarly, we do not know if a patient had declined their teleophthalmology or F2F appointment, however, this is being explored in future work. Finally, our evaluation pertained to healthcare provision in an exceptionally diverse population under the provisions of a universal healthcare system (NHS). Conclusions drawn must be considered in the context of an organised healthcare system from a single-provider single-payer system and may not be generalisable to regions without organised health systems.^29^

To our knowledge, there has been no similar large-scale investigation of factors associated with non-attendance within specialist ophthalmic care with which we can compare our findings. However, many of our findings echo those in other fields of healthcare.^30 31^ In ophthalmology, Eberly *et al* patients identifying as Asian and receiving Medicaid had fewer completed telemedicine visits while those identifying as black and with lower income demonstrated lower use of video for telemedicine, respectively.^32^ Such sociodemographic patterning in non-attendance is particularly concerning in ophthalmology given that many potentially blinding eye conditions are more common among those from the most socioeconomically deprived backgrounds and/or from ethnic minority groups.^33–35^ A key priority as telemedicine services mature will be the investigation of differential visual outcomes between patients undergoing F2F and telemedicine models of care.

In our study, the synchronous group was significantly younger than the asynchronous counterpart. This is likely to have resulted from older patients declining video consultations when offered. Furthermore, a large number of video consultations comprised assessments of patients with external (adnexal) eye conditions in particular benign eyelid lesions, who tend to be younger than the average ophthalmology patient.^36^ Our study demonstrates the association of self-reported black ethnicity and greater socioeconomic deprivation with lower attendance within synchronous models of care delivery as opposed to asynchronous. This may support a phenomenon gaining significant traction, ‘digital exclusion’, which refers to a sector of the population who suffer from inequitable access and limited competency to use Information and Communication Technologies.^37^ To probe this further, we investigated whether lack of access to broadband internet was associated with non-attendance using the DERI.^15^ The lack of association between the DERI and non-attendance in our study may have several possible explanations. The DERI refers to aggregate postcode-level data rather than at the individual level. In our predominantly urban-based population, small geographical areas likely contain populations with varying levels of access to digital services. Moreover, synchronous telemedicine is increasingly delivered using smartphone-based technology where internet access may be mediated through cellular signal.

While those from ethnic minority groups generally exhibited higher levels of non-attendance, especially in synchronous audiovisual appointments, opting not to self-report ethnicity was among the strongest associations. We hypothesised this to be an important determinant given previous evidence suggesting failure to self-report may be a surrogate of non-engagement with healthcare services.^23–25^ In our report, those with ‘unknown’ ethnicity were older, more socioeconomically deprived, had worse broadband access and greater levels of diabetes mellitus suggesting a group already at risk of worse health outcomes. While it is unclear whether targeted communication on the benefits of health engagement may improve attendance rates in this group, there are distinct advantages in improving the recording of ethnicity data through informing equity of access, clinical practice, supporting high quality research and service planning.^38^

Countering our hypothesis, we observed better attendance among those requiring an interpreter prior to the pandemic, however, this ‘protective effect’ was not present during the pandemic. Our findings may suggest that patients who have used the interpreter service in person demonstrate higher engagement with healthcare services, and this needs to be accounted for when planning interpretations support available in telemedicine services. Similarly, patients, who have been certified as sight-impaired, had lower non-attendance, possibly reflecting active engagement with the larger welfare apparatus by enrolling themselves in the system to receive sight-impaired status, a better understanding of the implications of sight loss and/or a fear of further deterioration. This finding should be somewhat reassuring to clinicians as this especially vulnerable group does not appear to need additional measures to ensure good attendance.

Ambitions towards digital healthcare transformation are such that teleophthalmology is likely to remain a core part of service delivery in countries with resources to implement it. The findings of our study concord with building evidence from other areas of healthcare of persistent limited engagement with healthcare services among certain groups, such as those from ethnic minority groups and those living in greater socioeconomic deprivation. Further investigation is warranted of how such differential engagement could be addressed—for example with improved, targeted communication on the benefits of improved engagement on the outcomes that matter to patients. We suggest that the development and maturation of telemedicine services should go hand in hand with investigations into differential health outcomes among underserved populations, as the best strategy to minimise the risk of amplifying and embedding pre-existing inequalities for patients.

## Data Availability

No data are available.
