# Supplementary material for: Determinants of non-attendance at face-to-face and telemedicine ophthalmic consultations
Source: Br J Ophthalmol. 2023 May 22;108(4):625–32. doi: 10.1136/bjo-2022-322389 (PMC10958256; doi:10.1136/bjo-2022-322389)
Supplement: Supplementary data [file bjo-2022-322389supp001.pdf]

Supplementary Materials

Determinants of non-attendance at face-to-face and telemedicine ophthalmic consultations

SK Wagner MD[1, 2, 3], LD Raja MD[1], M Cortina Borja PhD [4], J Huemer MD[1], RR Struyven MD[3, 5], PA Keane MD[1, 2, 3], K Balaskas MD[1, 2, 3], DA Sim PhD[1, 2], PBM Thomas PhD[1,2], JS Rahi PhD[2, 4, 6, 7], AL Solebo PhD[2, 4, ,6, 7], S Kang MD[1, 2, 3*]

[*] **Correspondence and reprint requests:** Swan Kang MBBS MBioch FRCOphth, Moorfields Eye Hospital NHS Foundation Trust, 162 City Road, London, United Kingdom. Tel: +44 207 253 3411 Email: swan.kang1@nhs.net

[1]Moorfields Eye Hospital NHS Foundation Trust, London, UK

[2]Institute of Ophthalmology, University College London, London, UK

[3]NIHR Biomedical Research Centre at Moorfields Eye Hospital NHS Foundation Trust and UCL Institute of Ophthalmology, London, UK

[4]Great Ormond Street Institute of Child Health, University College London, London, UK

[5]Centre for Medical Image Computing, Department of Medical Physics and Biomedical Engineering, University College London, London, UK

[6]Great Ormond Street Hospital NHS Foundation Trust, London, UK

[7]Ulverscroft Vision Research Group, University College London, London, UK

Methods

Index of Multiple Deprivation (IMD) was derived from the patient's residential postcode and cross-referenced to national data tables for 2019 provided by the UK Ministry of Housing, Communities & Local Government[1]. The Digital Exclusion Risk Index (DERI) covers twelve indicators across three equally-weighted domains - deprivation (33%), age (33%) and home broadband access and speed (33%) (full data available here: https://github.com/GreaterManchesterODA/Digital-Exclusion-Risk-Index)[2]. We suspected and statistically confirmed collinearity between the DERI and IMD (Pearson $r = 0.68$) and therefore opted to solely use the broadband component of the DERI. This comprises three indicators: i) the proportion of homes unable to receive a 30MBit/s connection, ii) the proportion of connections receiving less than 10MBit/s broadband and iii) the average download speed. The broadband component of the DERI did not correlate with IMD (r=0.04).

All analyses were conducted in R version 4.1.0 (R Core Team, 2021. R Foundation for Statistical Computing, Vienna, Austria). Multiple imputation was conducted using the `mice` package and visualisations were generated using the `ggplot` and `jtools` packages. Reporting is in line with the guidelines set by the Strengthening the Reporting of Observational Studies in Epidemiology (STROBE) and its extension, the REporting of studies Conducted using Observational Routinely-collected health Data (RECORD) statements (Checklist: Supplementary materials) [3,4].

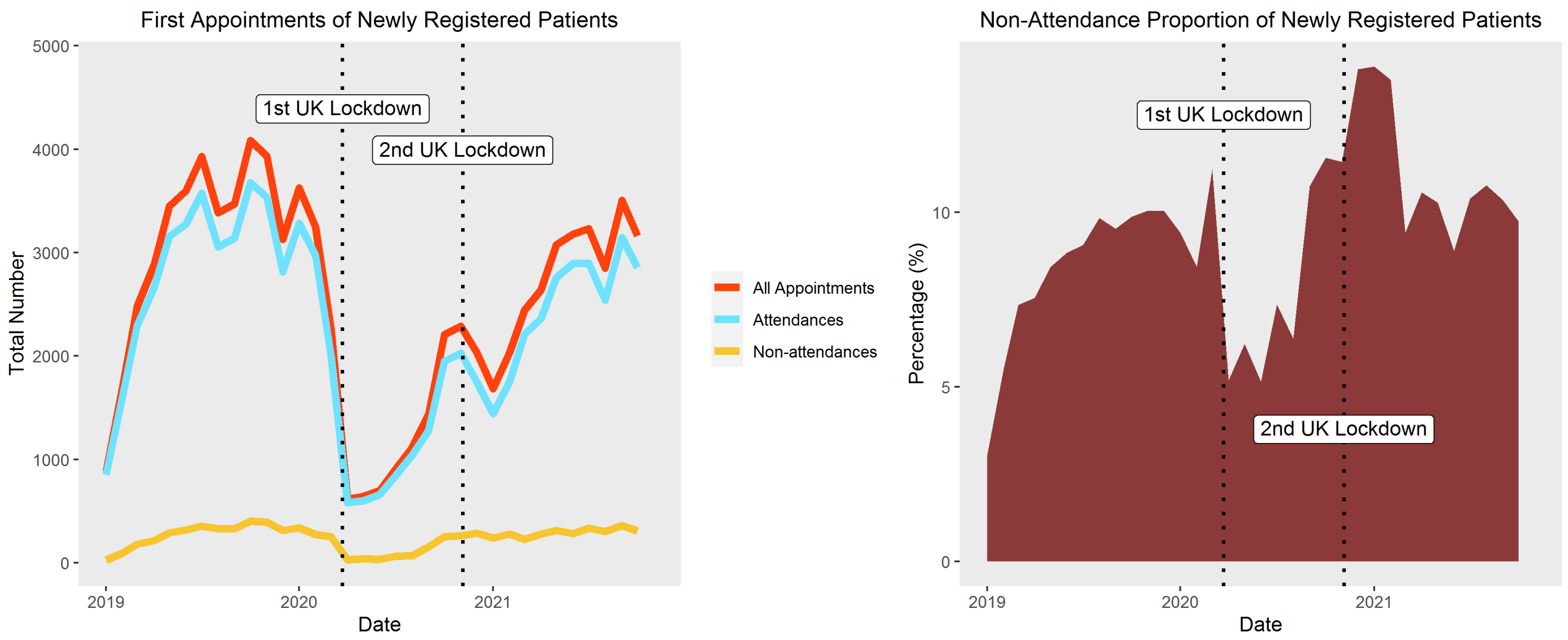

Supplementary Figure 1: Distribution of first appointments of newly registered patients by attendance status across the study period.

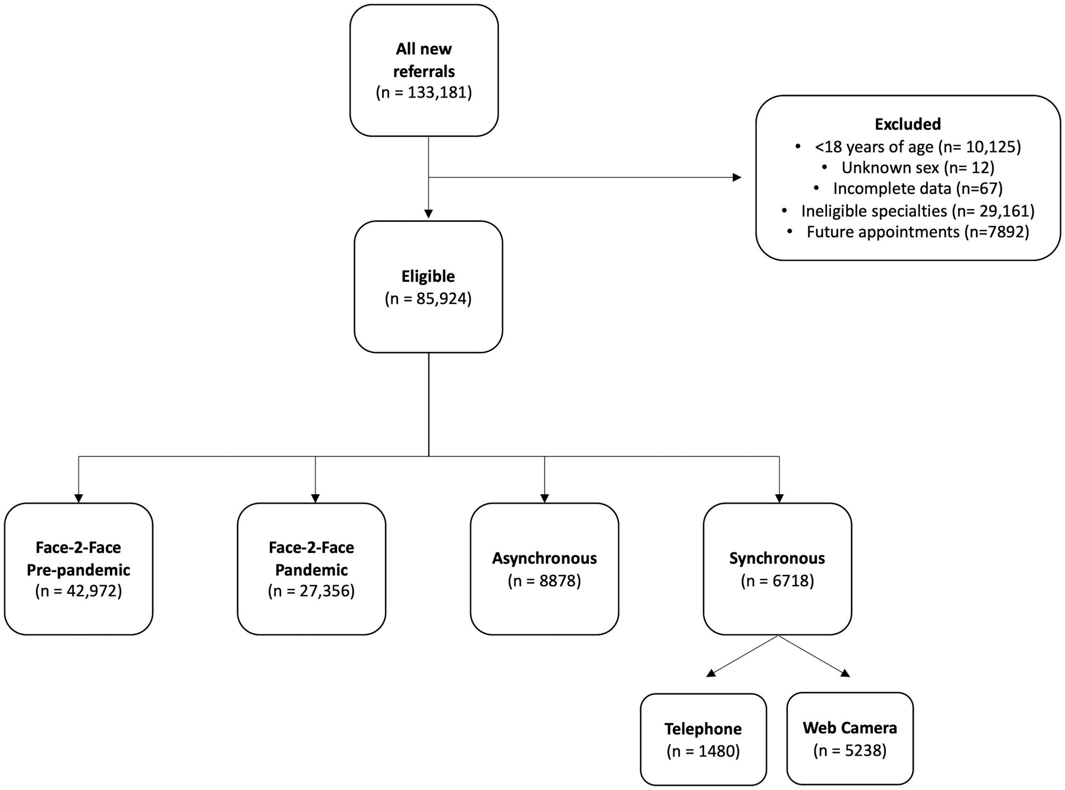

Supplementary Figure 2: Flow diagram illustrating the reasons for exclusion/ineligibility and division into mode of healthcare delivery. Pre-pandemic is defined as the period between January 1st 2019 and March 23rd 2021 inclusive, the date of the first UK lockdown. Pandemic is defined as March 24th 2021 to October 31st 2021 inclusive.

Supplementary Table 1: Pairwise comparisons of baseline characteristics by delivery mode.

Characteristic		F2F Pre-Pandemic	F2F Pandemic	Asynchronous	Synchronous – Telephone	Synchronous – Audiovisual
Sex	F2F Pre-Pandemic					
	F2F Pandemic	1				
	Asynchronous	**<0.001**	**0.0124**			
	Synchronous – Telephone	0.51	0.20	**0.0030**		
	Synchronous – Webcamera	**<0.001**	**<0.001**	**<0.001**	1	
Age	F2F Pre-Pandemic					
	F2F Pandemic	**<0.001**				
	Asynchronous	**<0.001**	**<0.001**			
	Synchronous – Telephone	**<0.001**	**<0.001**	**<0.001**		
	Synchronous – Webcamera	**<0.001**	**<0.001**	**<0.001**	**<0.001**	
Asian (South) ethnicity	F2F Pre-Pandemic					
	F2F Pandemic	**<0.001**				
	Asynchronous	**<0.001**	**<0.001**			
	Synchronous – Telephone	**0.0360**	1	1		
	Synchronous –Webcamera	**<0.001**	**0.0083**	1	1	
Black ethnicity	F2F Pre-Pandemic					
	F2F Pandemic	**<0.001**				
	Asynchronous	1	**<0.001**			
	Synchronous – Telephone	**0.0014**	1	**0.0016**		
	Synchronous – Webcamera	**<0.001**	0.08	**<0.001**	1	
Other ethnicity	F2F Pre-Pandemic					
	F2F Pandemic	**<0.001**				
	Asynchronous	**<0.001**	**<0.001**			
	Synchronous – Telephone	**<0.001**	**<0.001**	1		
	Synchronous – Webcamera	**<0.001**	**<0.001**	**<0.001**	**<0.001**	
White ethnicity	F2F Pre-Pandemic					
	F2F Pandemic	**<0.001**				

	Asynchronous	**<0.001**	1			
	Synchronous – Telephone	**<0.001**	**<0.001**	**<0.001**		
	Synchronous – Webcamera	**<0.001**	0.33	1	**<0.001**	
Unknown ethnicity	F2F Pre-Pandemic					
	F2F Pandemic	**<0.001**				
	Asynchronous	**<0.001**	**<0.001**			
	Synchronous – Telephone	**<0.001**	**<0.001**	**<0.001**		
	Synchronous – Webcamera	**0.0019**	**<0.001**	**<0.001**	**<0.001**	
SES	F2F Pre-Pandemic					
	F2F Pandemic	1				
	Asynchronous	**0.0028**	**0.0271**			
	Synchronous – Telephone	**0.0013**	**0.0035**	0.37		
	Synchronous – Webcamera	**<0.001**	**<0.001**	**<0.001**	0.15	
Non-attendance	F2F Pre-Pandemic					
	F2F Pandemic	**<0.001**				
	Asynchronous	**<0.001**	**0.0051**			
	Synchronous – Telephone	1	1	0.19		
	Synchronous – Webcamera	**<0.001**	**<0.001**	**<0.001**	**0.0207**	

Supplementary Table 2. Baseline characteristics of the cohort failing to self-report ethnicity.

	**Characteristic**	**Unknown N=53,787**	**Reported N=32,137**	***p*-value**
**Sex n (%)**	Female	29,551 (54.9%)	17,185 (53.5%)	**<0.001**
**Age median (IQR)**	Years	56 (14.5)	53 (15.5)	**<0.001**
**SES[2] median (IQR)**	Decile (1=most deprived)	5 (2)	5 (2)	**0.016**
**Broadband Access[3] median (IQR)**	Index (1=most at risk of digital exclusion)	3.40 (0.2)	3.41(0.2)	**<0.001**
**Interpreter n (%)**	Yes	451 (0.8%)	281 (0.9%)	0.61
**Diabetes n (%)****	Yes	2,992 (5.6%)	1,200 (3.7%)	**<0.001**
**Time of Appointment n (%)**	Early Morning	24,015 (44.6%)	14,548 (45.2%)	**<0.001**
	Late Morning	4,749 (8.8%)	2,711 (8.4%)	
	Early Afternoon	19,748 (36.7%)	11,275 (35.1%)	
	Late Afternoon	5,275 (9.8%)	3,603 (11.2%)	
**Sight-Impaired n (%)**	Yes	318 (0.6%)	172 (0.5%)	0.31
**Previous cancellation n (%)**	No	44,067 (81.9%)	28,195 (87.7%)	**<0.001**
	By hospital	7,037 (13.1%)	2,743 (8.5%)	
	By patient	2,683 (5.0%)	1,199 (3.7%)	
**Specialty n (%)**	Adnexal	5,484 (10.2%)	1,538 (4.8%)	**<0.001**
	Cataract	10,034 (18.7%)	5,085 (15.8%)	
	General	18,559 (34.5%)	16,654 (51.8%)	
	Glaucoma	8,672 (16.1%)	3,304 (10.3%)	
	Medical Retina	11,038 (20.5%)	5,556 (17.2%)	
**Attendance status n (%)**	Non-attendance	5,948 (11.1%)	2,340 (7.3%)	**<0.001**

Supplementary Table 3: Adjusted odds ratios for non-attendance derived from multivariable logistic regression stratified by delivery mode with multiple imputation (predominantly ethnicity, but also SES and Broadband)

		Pre-pandemic F2F		Pandemic F2F		Asynchronous		Synchronous (telephone)		Synchronous (audiovisual)	
		**OR (95% CI)**	***p*-value**	**OR (95% CI)**	***p*-value**	**OR (95% CI)**	***p*-value**	**OR (95% CI)**	***p*-value**	**OR (95% CI)**	***p*-value**
**Age**	(decade)	0.92 (0.90-0.94)	**<0.001**	0.90 (0.88-0.92)	**<0.001**	0.90 (0.86-0.95)	**<0.001**	0.83 (0.73-0.95)	**0.0063**	1.05 (0.98-1.13)	0.14
**Sex**	Female	**Reference**		**Reference**		**Reference**		**Reference**		**Reference**	
	Male	1.17 (1.09-1.25)	**<0.001**	1.11 (1.02-1.20)	**0.0119**	1.27 (1.11-1.45)	**<0.001**	1.26 (0.88-1.82)	0.20	1.31 (1.05-1.63)	**0.0161**
**Ethnicity**	White	**Reference**		**Reference**		**Reference**		**Reference**		**Reference**	
	Asian (South)	1.05 (0.91-1.21)	0.49	1.14 (0.91-1.43)	0.23	1.44 (1.01-2.04)	**0.0416**	1.49 (0.48-4.57)	0.47	2.43 (1.32-4.48)	**0.0052**
	Black	1.28 (1.08-1.51)	**0.0058**	1.69 (1.30-2.20)	**<0.001**	1.02 (0.76-1.39)	0.88	2.58 (0.70-9.46)	0.14	4.62 (2.27-9.42)	**<0.001**
	Other	1.37 (1.23-1.53)	**<0.001**	2.04 (1.66-2.50)	**<0.001**	2.41 (1.90-3.06)	**<0.001**	2.67 (1.03-6.93)	**0.0440**	1.67 (1.00-2.79)	**0.0050**
**SES (greater deprivation)**	Per decile decrease	1.11 (1.10-1.13)	**<0.001**	1.08 (1.06-1.10)	**<0.001**	1.07 (1.04-1.11)	**<0.001**	1.03 (0.95-1.12)	0.47	1.16 (1.10-1.23)	**<0.001**
**Broadband**	Index	0.94 (0.86-1.01)	0.10	0.99 (0.90-1.09)	0.84	0.94 (0.80-1.11)	0.46	0.81 (0.52-1.26)	0.34	1.06 (0.84-1.34)	0.56
**Interpreter**	No	**Reference**		**Reference**		**Reference**		**Reference**		**Reference**	
	Yes	0.52 (0.34-0.79)	**0.0023**	1.09 (0.72-1.67)	0.68	1.26 (0.62-2.54)	0.52	2.55 (0.75-8.73)	0.13	0.70 (0.10-5.25)	0.73
**Diabetes mellitus**	No	**Reference**		**Reference**		**Reference**		**Reference**		**Reference**	
	Yes	0.88 (0.75-1.04)	0.13	0.58 (0.48-0.70)	**<0.001**	0.53 (0.41-0.67)	**<0.001**	1.48 (0.31-7.08)	0.62	*	0.50

**Subspecialty**	Adnexal	**Reference**		**Reference**		*		**Reference**		**Reference**	
	Cataract	1.46 (1.24-1.72)	**<0.001**	1.00 (0.86-1.17)	0.98	*		1.20 (0.55-2.61)	0.65	1.16 (0.68-1.97)	0.59
	General	1.31 (1.14-1.52)	**<0.001**	0.45 (0.38-0.51)	**<0.001**	*		1.38 (0.62-3.08)	0.43	0.55 (0.44-0.71)	**<0.001**
	Glaucoma	1.64 (1.38-1.95)	**<0.001**	0.96 (0.80-1.15)	0.65	**Reference**		*		*	
	MR	2.08 (1.74-2.37)	**<0.001**	1.40 (1.21-1.61)	**<0.001**	2.39 (2.06-2.77)	**<0.001**	*		*	
**Registered sight-impaired**	No	**Reference**		**Reference**		**Reference**		**Reference**		**Reference**	
	Yes	0.34 (0.18–0.64)	**<0.001**	0.50 (0.28-0.90)	**0.0208**	0.59 (0.14-2.57)	0.49	*	*	*	
**Appointment time**	Early afternoon	**Reference**		**Reference**		**Reference**		**Reference**		**Reference**	
	Early morning	1.09 (1.01-1.17)	**0.0196**	1.05 (0.97-1.15)	0.22	1.15 (0.97-1.36)	0.11	0.83 (0.51-1.35)	0.44	0.92 (0.71-1.18)	0.50
	Late morning	0.72 (0.61-0.85)	**<0.001**	0.97 (0.83-1.13)	0.68	1.03 (0.82-1.28)	0.82	0.59 (0.26-1.34)	0.21	0.74 (0.51-1.08)	0.12
	Late afternoon	0.63 (0.54-0.74)	**<0.001**	0.58 (0.47-0.73)	**<0.001**	1.18 (0.95-1.46)	0.13	1.66 (1.08-2.56)	**0.0221**	0.59 (0.41-0.83)	**0.0028**
**Previous cancellation**	No	**Reference**		**Reference**		**Reference**		**Reference**			
	By the hospital	1.32 (1.19-1.47)	**<0.001**	1.91 (1.72-2.12)	**<0.001**	2.08 (1.71-2.52)	**<0.001**	0.40 (0.21-0.75)	**0.0042**	2.19 (1.61-2.98)	**<0.001**
	By the patient	1.88 (1.63-2.15)	**<0.001**	1.84 (1.56-2.18)	**<0.001**	1.59 (1.26-2.01)	**<0.001**	1.37 (0.65-2.91)	0.41	1.63 (0.90-2.98)	0.11

*Either no cases or very few leading to unstable estimates.

F2F: Face-to-face, OR: Odds ratio, SES: Socioeconomic status, MR: Medical retina.

References

1 Ministry of Housing, Communities, Local Government. English indices of deprivation 2019. 2019.https://www.gov.uk/government/statistics/english-indices-of-deprivation-2019 (accessed 24 Feb 2023).

2 A new tool: the Digital Exclusion Risk Index. Good Things Foundation. 2021.https://www.goodthingsfoundation.org/a-new-tool-in-your-toolbox-the-digital-exclusion-risk-index/ (accessed 24 Feb 2023).

3 Benchimol EI, Smeeth L, Guttmann A, *et al.* The REporting of studies Conducted using Observational Routinely-collected health Data (RECORD) Statement. *PLoS Med* 2015;**12**. doi:10.1371/journal.pmed.1001885

4 von Elm E, Altman DG, Egger M, *et al.* The Strengthening the Reporting of Observational Studies in Epidemiology (STROBE) statement: guidelines for reporting observational studies. *PLoS Med* 2007;**4**. doi:10.1371/journal.pmed.0040296
